# Rapid protocol for visualization of rust fungi structures using fluorochrome Uvitex 2B

**DOI:** 10.1186/s13007-015-0096-0

**Published:** 2015-12-11

**Authors:** Sheshanka Dugyala, Pawel Borowicz, Maricelis Acevedo

**Affiliations:** Department of Plant Pathology, North Dakota State University, Dept. 7660, PO Box 6050, Fargo, ND 58108-6050 USA; Department of Animal Sciences, North Dakota State University, Dept. #7630, PO Box 6050, Fargo, ND 58108-6050 USA

**Keywords:** Wheat, Leaf rust, Fungus, *Puccinia triticina*, Uvitex 2B, Histology, Staining, Fluorescence microscopy, Infection structures, Host-pathogen interaction

## Abstract

**Background:**

Histological examination using fluorochromes is one of the standard methods for observation of microorganisms in tissues and other compartments. In the study of fungi, especially those that cannot be cultured in axenic media such as biotrophic fungi, histological examination of processes associated with the fungal growth, differentiation, infection and other cellular functions can lead to the better understanding of host-parasite interactions. Fluorescence microscopy coupled with Fluorochrome Uvitex 2B have been extensively utilized to study rust fungi structures and host-pathogen interactions. In this study, we report development of a rapid staining protocol of the rust fungus *Puccinia triticina* using fluorochrome Uvitex 2B. The newly developed rapid procedure was compared with a standard staining technique to observe *in planta* fungal infection structures development during the wheat—*Puccinia triticina* interaction.

**Results:**

While significantly reducing the time for staining, the rapid protocol described here was equally efficient or better compared to standard procedure in detecting fungal infection structures using Uvitex 2B. In the rapid staining procedure, pre-heating of the stain increased efficiency to detect all the infection structures including haustoria with highly reduced background noise from plant tissue.

**Conclusion:**

This staining process described here is simple and quick. It can be completed in 4 h, which is of 6 times faster than the standard Uvitex 2B staining procedure.

**Electronic supplementary material:**

The online version of this article (doi:10.1186/s13007-015-0096-0) contains supplementary material, which is available to authorized users.

## Background

Histology is considered as one of the most appropriate technique to detect fungus and its infection structure development in plant and animal systems. The histological examination of fungal structure morphology and development during the infection process has offered valuable information to mycologists and plant pathologists for diagnosis, identification, classification of pathogenic microorganisms and their interaction with host and non-host plants specially when dealing with biotrophic fungi such as rust pathogens [1, 2]. In recent years a new interest in histology has been prompted by the availability of more powerful microscopes and the interest on connecting molecular and functional genomics discoveries to physical components. The fluorochromes Calcoflour White and Uvitex 2B and fluoresce microscopy have been extensively utilized to study rust fungi structures and host-pathogen interactions [3–14]. Both fluorochromes, Uvitex 2B and Calcoflour White, bind to the chitin present in the fungal body wall allowing fungal detection [15, 16]. However, Calcoflour White fades away quickly when exposed to light, including it’s specific excitation wavelengths and stains plant structures containing cellulose making it difficult to differentiate fluorescence between the plant structures like trichomes, and fungal structures [14, 17, 18]. Therefore, Uvitex 2B has been more widely used to study rust fungi infection structures development in host and non-host interactions [1, 12, 19–21]. Currently used staining procedures are time consuming, and involve numerous chemical reagents including chloroform, methanol, lactophenol, ethanol, and NaOH. The procedure for fixation and staining of rust fungi as described by Kuck 1981 [23] has been the most widely used for staining rust fungi. This protocol requires 12–18 h for fixing and clearing of specimens, depending on the stage of the plant development (adult or seedling) and staining requires an additional 5 h. The length of the protocol and numerous steps has limited its applicability when dealing with large number of specimens or when downstream molecular biology applications require intact cells and cell components such as nucleic acids.

The objective of the current study was to develop a rapid and less complex staining procedure that can be utilized to evaluate all infection structures during *Puccinia triticina* infection of wheat. We describe a rapid Uvitex 2B staining protocol that can be utilized for easy and comprehensive observation of the wheat leaf rust pathogen *Puccinia triticina* fungal structures *in planta* using wide-field fluorescence and structured illumination techniques. The newly developed rapid procedure was compared with the Uvitex 2B standard staining technique to observe *in planta* fungal infection structures development during wheat—*Puccinia triticina* interaction.

## Results and discussion

### Rapid protocol development and comparison to standard protocol

**Fig. 1:**
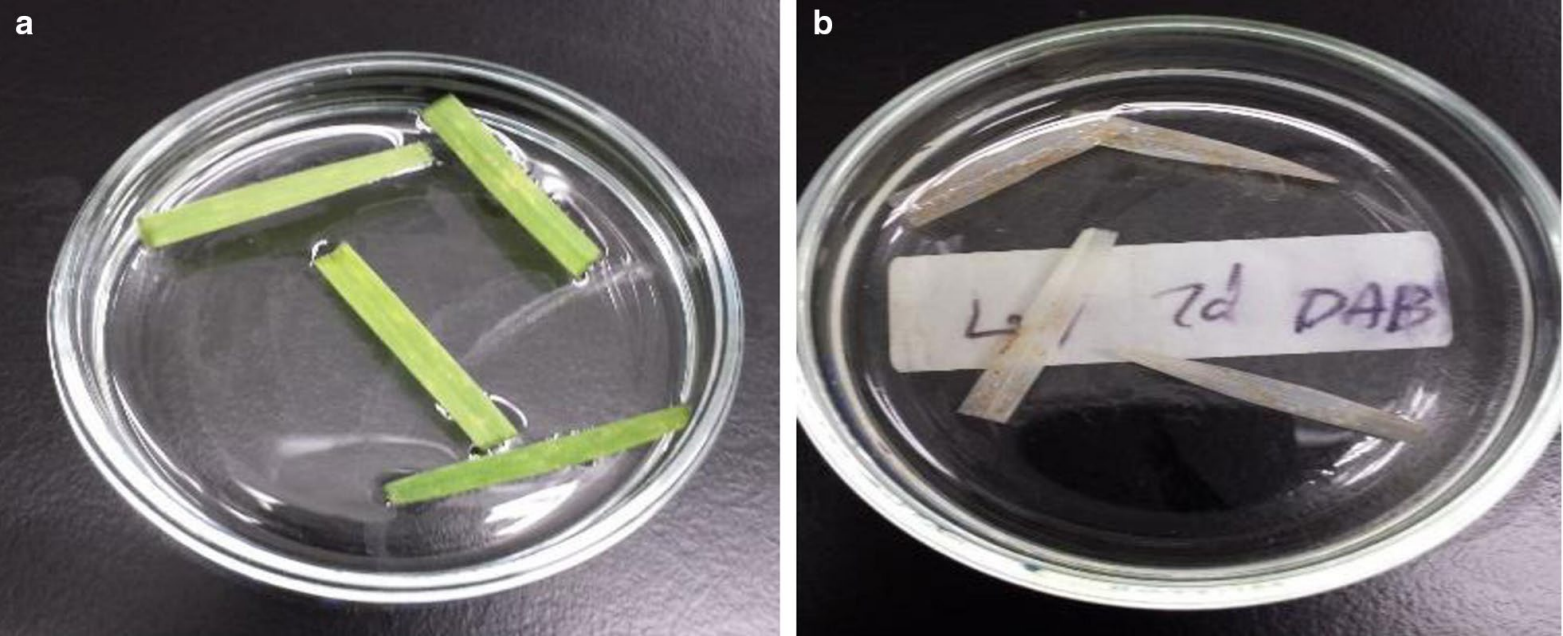
Conditions and characteristics. **a** Wheat cultivar Thatcher leaf tissue collected and placed directly into Farmer’s fixative immediately after collection. **b** Fixed and cleared specimens after 60 min in Farmer’s fixative

Infected leaves of susceptible wheat cultivar Thatcher were sampled at various time points, stained using both staining methods (rapid and standard), and observed under the fluorescence microscope. In the rapid staining procedure, clearing and fixing steps were combined into one single step by replacing chloroform–methanol and lactophenol-ethanol, with ethanol:acetic acid (3:1 v/v) (Fig. 1a, b). Since Farmer’s fixative only requires a short period of time to clear and fix specimens, the 18–24 h period required for clearing and fixing in the standard procedure was reduced to just 1 h. By using Farmer’s fixative instead of lactophenol-ethanol no additional ethanol, 0.1 N NaOH or water washing steps were required as recommended in the standard Uvitex 2B method [8–14, 24].

**Fig. 2:**
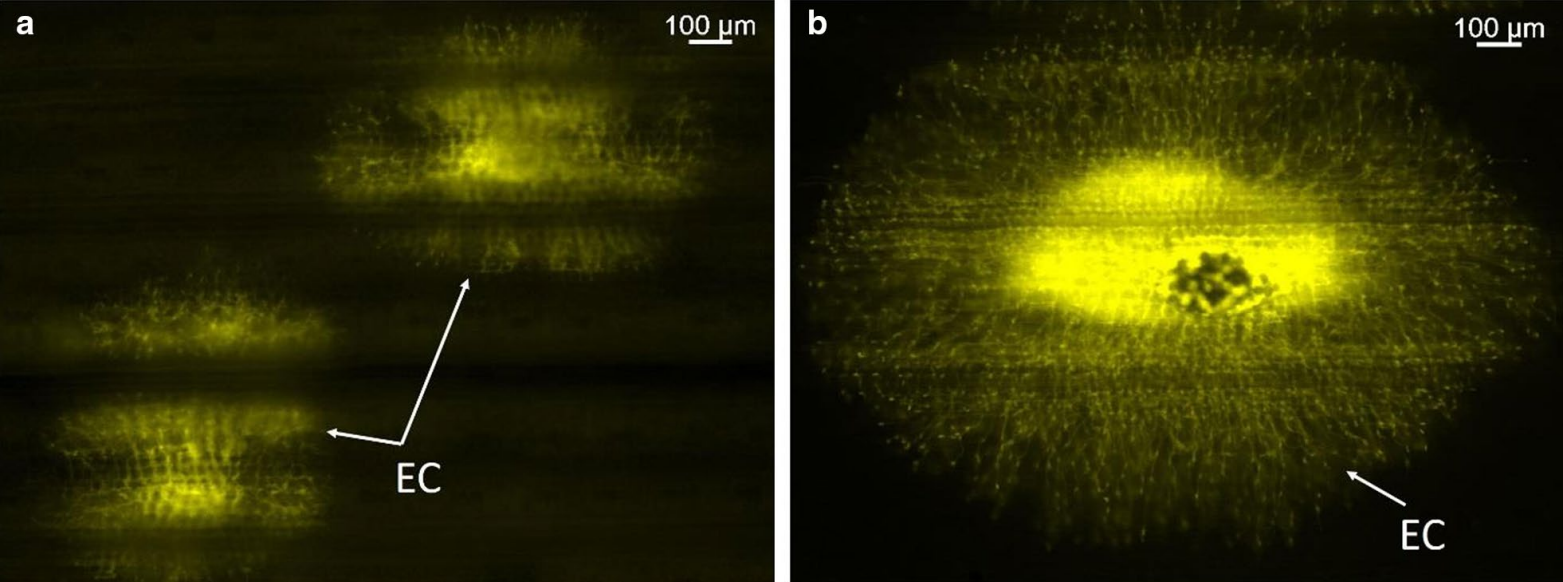
Comparison of pre-staining incubation time. Leaves of seedling stage plants of cultivar Thatcher infected by *Puccinia triticina* were stained with fluorochrome Uvitex 2B and examined under an Axio Imager M2 Zeiss Research epifluorescence microscope. **a** Under-stained *P. triticina* colony in leaf tissue treated with a 5 min pre-staining incubation. **b** Brighter *P. triticina* colony after a 10 min pre-staining treatment in 0.1 M Tris–HCl. Established colony (*EC*)

Soaking of specimens in 0.1 M Tris–HCl (pH 8.5) before staining step was found to be necessary for effective staining. Specimens were not stained when not soaked in Tris-HCLl or poorly stained, when soaked for only 5 min irrespective of staining temperature (Fig. 2a). Specimens were stained uniformly when soaked in Tris–HCl for at least 10 min (Fig. 2a). No apparent staining advantage was noticed with increase pre-stain soaking time (15 or 30 min) compared to 10 min. Overall, specimens stained with pre-heated (65 °C for 5 min) 0.3 % Uvitex 2B appeared bright and haustoria were clearly visible compared to staining solution at room temperature. In addition, a reduction of non-specific staining was achieved. Based on the above results, fixing and clearing for only 1 h, pre-stain soaking in 0.1 M Tris–HCl for 10 min and staining with pre-heated 0.3 % Uvitex 2B for 5 min at 65 °C were identified as optimum conditions for visualization of fungal structures *in planta* using fluorescence microscopy.

**Fig. 3:**
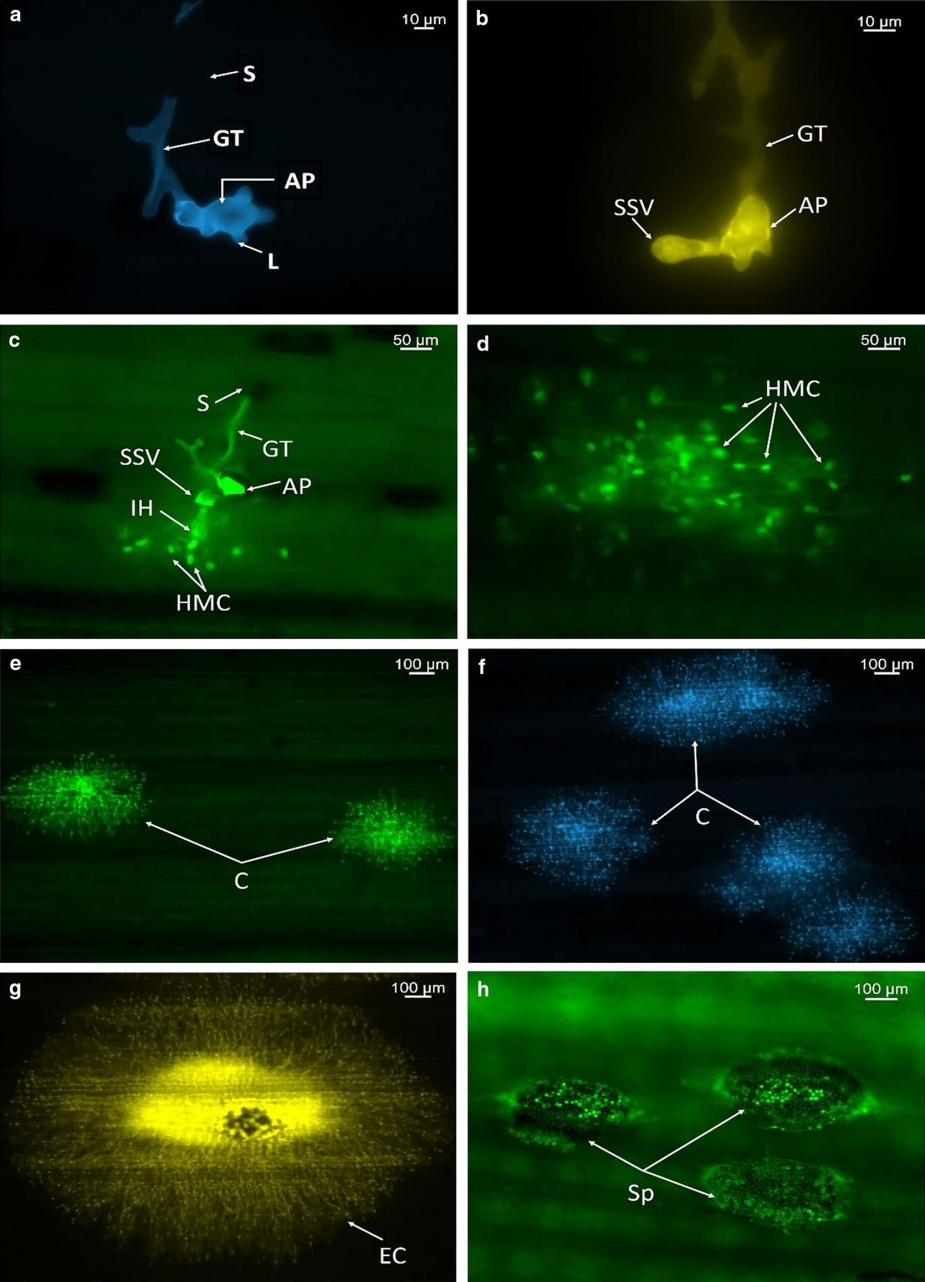
*Puccinia triticina* infection on susceptible wheat cultivar Thatcher. Seedling stage plant specimens examined under an Axio Imager M2 Zeiss Research epifluorescence microscope (**a**–**h**). **a** Germinated spore (*S*) 4 hpi, with branching germ tube (*GT*) and appressorium (*AP*) with lobes (*L*). **b** At 12 hpi, infection peg formed from appressorium and sub-stomatal vesicle (*SSV*) formation from infection peg. **c** At 12 hpi, Formation of infection hyphae (*IH*) from infection peg. **d** At 18 hpi, haustorial mother cell (*HMC*) produced a penetration peg that perforated through mesophyll cell wall and formed a haustorium in the host cell. **e**, **f** At 24 –96 hpi, increase number of haustorial mother cells, and colony development (*C*). **g**, **h** At 7 dpi, established colony (*EC*) and sporulation (*SP*)

**Fig. 4:**
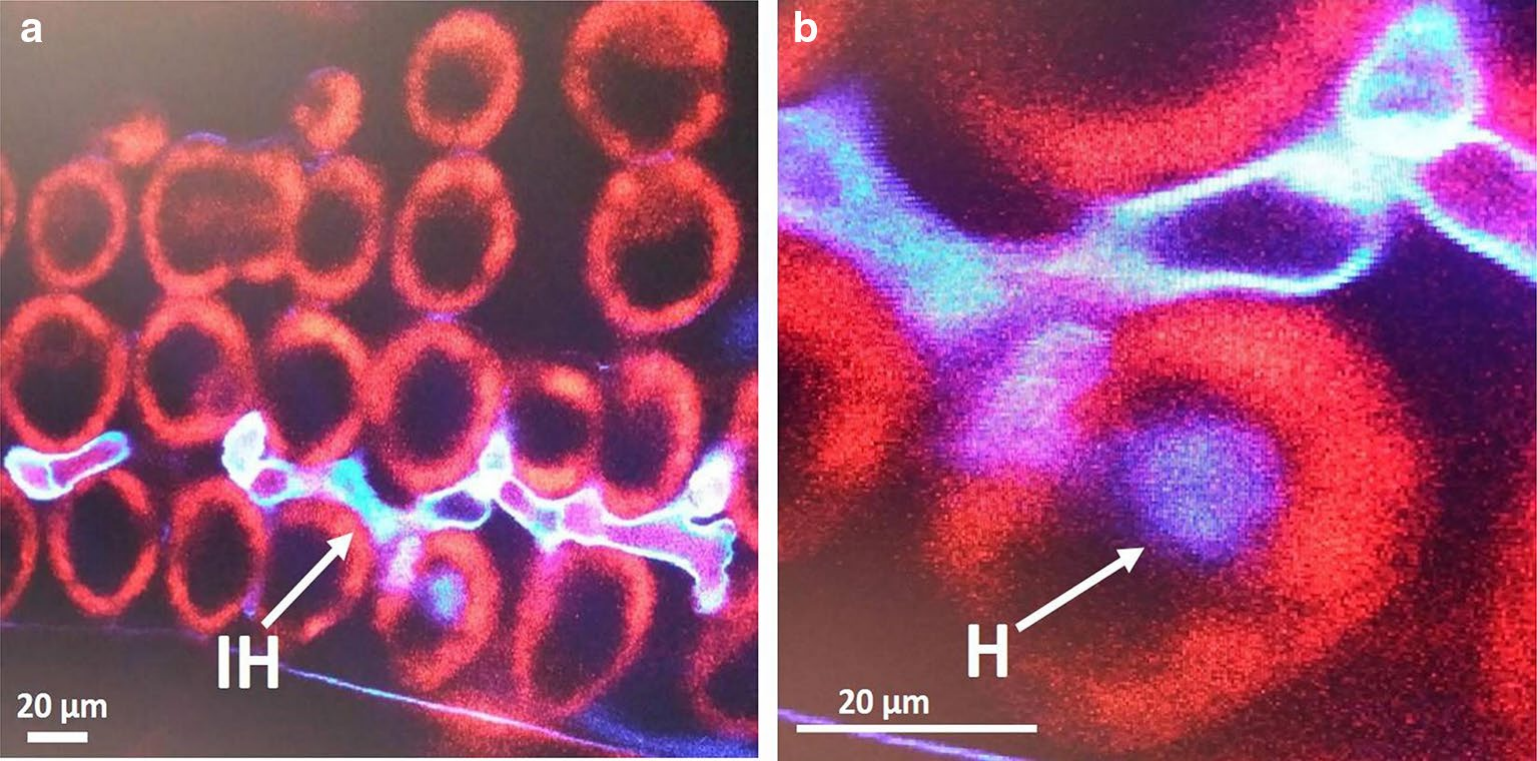
Confocal microscopy of haustoria from wheat-*Puccinia triticina* infected tissue. Seedling stage plant specimens examined under laser scanning Alexa Fluor 488 confocal microscope (**a**, **b**). **a** At 20 hpi, formation of intercellular primary infection hyphae (*IH*), differentiated into haustorial mother cells (HMC) once in contact with mesophyll cells of host. **b** Haustorial mother cells penetrated through the host mesophyll cells wall to form haustorium (*H*)

Using both Uvitex 2B methods (standard and rapid), evaluation of fungal structures on Thatcher seedling plants after inoculation with *P. triticina* race THBL urediniospores was possible. Spore germination and germ tube was observed within 2 h after spraying of inoculum onto seedling plants leaf surface (Fig. 3a–h). Germinating urediniospores in many cases had two germ tubes but only one of the germ tubes differentiated into an appressorium (Fig. 3a). Germ tube branching was observed as previously described for *P. triticina* [25]. At 4 h post incubation (hpi), appressorium with 3–4 lobes were observed in the stomata on the leaf surface (Fig. 3b) After the appressorium was formed, other infection structures including infection peg, sub-stomatal vesicle, and infection hyphae were observed at 12 hpi (Fig. 3c and Additional file 1). Haustorial mother cells (HMC) were observed by 16–18 hpi (Fig. 3d and Additional file 2). In later time points (48, 72 and 96 hpi) an increase number of HMC, and further colony development was observed (Fig. 3e–g). Finally, by 7 days post inoculation (dpi), completely established colonies with sporulation were observed (Fig. 3h). Matured and newly formed spores were observed in established colonies (Fig. 3h). Despite not able to observe haustoria under wide-field fluorescence microscope at any of the tested magnifications, haustoria were clearly observed inside the plant cell by 20 hpi, with laser scanning confocal microscope at 40× magnification (Fig. 4a, b).

**Fig. 5:**
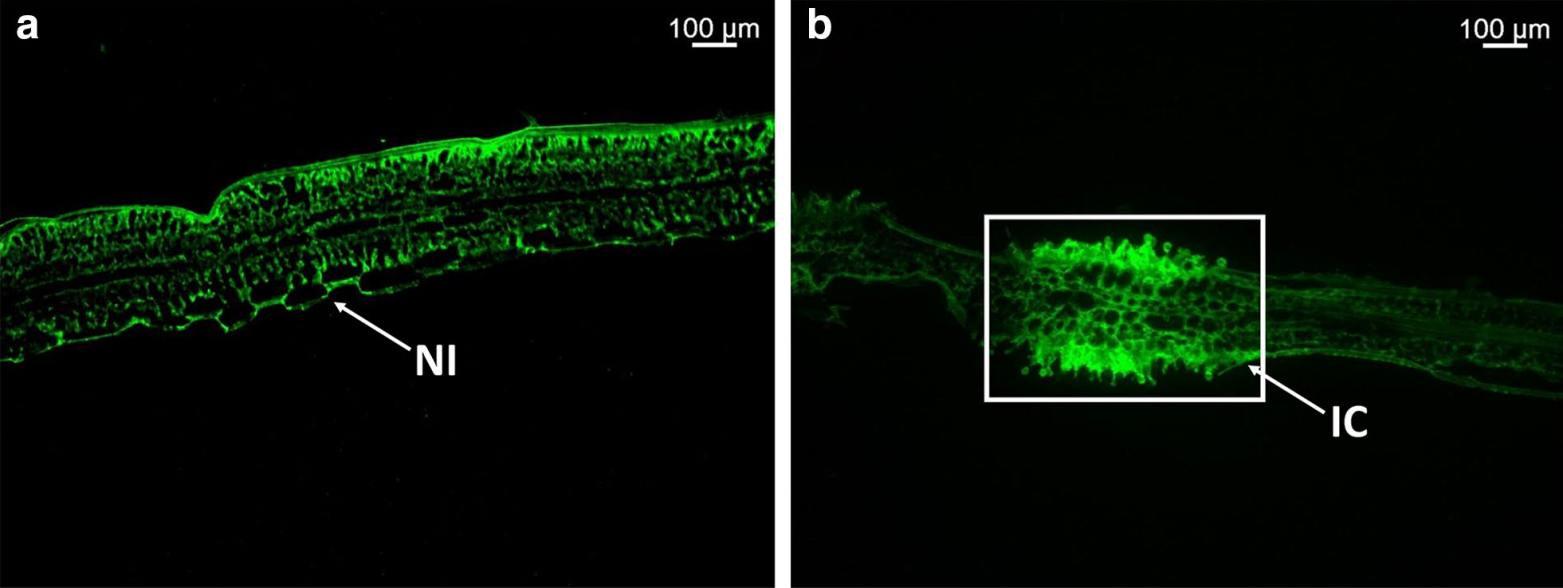
Sample preparation for laser capture microdissection of infected wheat cells. Wheat leaf specimens examined under an Axio Imager M2 Research epifluorescence microscope (**a**, **b**). **a** Longitudinal section of non-inoculated wheat leaf stained with Uvitex 2B. **b** Longitudinal section of wheat leaf inoculated with *P. triticina*. Within *box*, infection site with uredinium was excised via LCM and collected for RNA extraction of host and pathogen cells

In a small preliminary experiment, we used the rapid Uvitex 2B to stain *P. triticina* inoculated wheat leaf samples prepared for Laser Capture Microdissection and RNA extraction of host and pathogen from 500 dissected cells. Using this method, we were able to successfully identify infected cells (Fig. 5a, b) for excision. Total RNA was then extracted from the excised infected cells and used in the amplification of the *P. triticina* ubiquitin gene. The RNA amplification threshold cycles for Ubiquitin of the LCM specimens were comparable to those obtained from diluted RNA specimens extracted from full wheat leaf tissue without staining or LCM preparation (Acevedo et al. unpublished).

## Conclusions

We have developed a rapid and effective way to stain wheat leaf tissue infected with the fungal pathogen *P. triticina* and were able to clearly observe and distinguish all fungal infection structures *in planta* in a time course experiment. In the newly developed rapid Uvitex 2B protocol there is only a single step involved for fixing and clearing the specimen and it reduced the time from 18 to 24 h to only 1 h. By pre-heating the fluorochrome Uvitex 2B a brighter staining of fungal structures and better contrast against plant cell was obtained.

Currently developed rapid staining protocol has several advantages over the standard Uvitex 2B staining protocol that has been used to study fungal infection process *in planta*. First, staining the specimens using this rapid staining procedure is fast and simple, fixation and staining can be completed within 1 and 3 h, respectively. Second, the pre-heating of the Uvitex 2B before the staining step allows for better detection of fungal structures including haustoria (when coupled with confocal microscopy) and provides a clear differentiation between host and fungal structures. Third, with this rapid staining procedure the number of chemical reagents is reduced which results in a lower cost per specimen.

Moreover, due to the short time required from collection to observation of specimens and rapid specimen fixation step, the rapid Uvitex 2B staining protocol is suitable for use in the preparation of samples for plant-cell isolations maintaining nucleic acid integrity. The ability to amplify RNA from excised cells from the Uvitex 2B stained samples demonstrates that the rapid staining protocol does not affect quality of RNA making this protocol suitable for specific cell gene expression studies.

Furthermore, the rapid staining procedure has been successfully applied to other rust pathogen-host and non-host interaction including *Puccinia graminis* f. sp. *tritici*-*Hordeum vulgare, P. triticina*—*Triticum turguidum* subsp. durum, P*. triticina*—*H. vulgare* and *Puccinia coronata* f. sp. *avenae*—*Avena sativa* (data not shown). Future work will determine if the rapid staining protocols can be utilized in other pathosystems outside of the cereal rusts.

## Methods

### Plant material

Wheat cultivar Thatcher [26], susceptible to most of the known wheat leaf rust races was used in this study. Seedling were grown in flats in a rust free greenhouse at (18–21 °C day, 16–18 °C night, 16 h photoperiod) until second leaf stage was reached (primary leaf fully expanded).

### Experimental design

The experimental design was a completely randomized design, with each cell containing three seedlings considered as an experimental unit replication. Three replications were used for microscopic observations at 2 h post incubation (hpi), 4, 6, 12, 18, 24, 48, 72, 96 hpi and 7 days post inoculation (dpi) for both staining methods (rapid and traditional staining methods).

### Pathogen inoculations

A single spore isolate of the wheat leaf rust pathogen, *Puccinia triticina,* race THBL was used for inoculation. Fresh spores were utilized and urediniospores concentration was adjusted to 6.2 × 10^5^ spores per ml in Soltrol 170 oil. Inoculations were done using a spray inoculator at secondary leaf stage. After inoculation plants were allowed to dry for 30 min before incubation in a mist chamber with a 100 % relative humidity (RH) for 16 h in dark and then transferred to a greenhouse compartment kept at 18–22 °C (day) and 18–20 °C (night) with a 16 h photoperiod, and 80 % RH.

### Sample preparation

For all microscopic observations two 3-cm leaf pieces from the central portion of primary leaf were collected in a time course at 6, 24, 48, 72, 96 hpi and 7 dpi. After cutting the leaf tissue all specimens were placed in a plastic centrifuge tube and placed in an ice bath until further processing.

### Development of rapid staining protocol

To develop the rapid staining protocol using Uvitex 2B, specimens collected from 24 hpi were used as all the infection structures could be observed at this time point. Fixing and clearing was performed in a single step as previously described by Moldenhauer et al. [12], with some modifications. In short, fixing and clearing involved placing each specimen in a 60 mm glass petri dish containing 15 mL Farmer’s fixative (ethanol:acetic acid 3:1 v/v) and shake for 1 h at 140 rpm on an orbital shaker at room temperature (approx. 21 °C). Specimens were washed with deionized water twice for 10 min each. To determine the optimal conditions for rapid staining of specimens, multiple combination of pre-stain soaking, staining and incubation time and temperature, were tested (Table 1). After staining, specimens were washed once with deionized water for 10 min and stored in 50 % glycerol until specimens were mounted on microscopic slides.

**Table 1:** Combinations of staining temperature and pre-staining incubation time tested to determine best conditions to differentiate fungal structures when staining wheat leaf specimens inoculated with *P. triticina*

Staining temperature	Room temperature (min)	65 °C (min)
Pre-staining incubation time	0	0
	5	5
	10	10
	15	15
	30	30

### Standard staining procedure

To determine if the new rapid protocol provided reliable data, equal or better than the previously described Uvitex 2B staining protocols, a second set of specimens from the experiment described above were stained using the standard protocol which is a modified version of Rohringer et al. [26] as described by Moldenhauer et al. [12]. This protocol was used as the standard as it has been the most widely used procedure in previously published histological evaluation research using Uvitex 2B [3–14]. Briefly, infected leaves were fixed and cleared with lactophenol-ethanol or chloroform–methanol mostly by boiling method, and then washed with a series of different solutions including ethanol, NaoH, and water before and after staining the specimens.

### Microscopic observations

Three primary leaves sections (2 cm each) from each of the three replicates were collected and six slides (two slides per each primary leaf) were mounted for microscopic observations. A total of 10–15 infection units were observed per each slide per time point and images of representative infection sites were acquired. Specimens were examined under wide-field fluorescence using an Zeiss Axio Imager M2 research epifluorescence upright microscope with an excitation filter BP379-401; chromatic beam splitter FT 420; emission 435–485; using AxioVision rel. 4.8 software. All observations were carried at 5×, 20× and 40×. For confocal microscopy and haustoria observation, specimens were observed using Zeiss Axio Observer Z1 inverted microscope with LSM700 laser scanning unit, 405 solid state laser at 40× magnification. Staining procedures were evaluated for their efficiency in visualizing pathogen infection structures including spore, germ tube, appressorium, sub-stomatal vesicle (SSV), haustorial mother cell (HMC) and haustorium; and background noise from staining host structures.

## Additional files

10.1186/s13007-015-0096-0 3D view of wheat leaves infected with *Puccinia triticina* (12 hpi). The video shows seven-day-old seedling plants of susceptible cultivar Thatcher infected with *P. triticina*. Specimen was stained using the Uvitex 2B rapid staining method and imaged at 12 using confocal microscopy and analyzed with Contour Surface Module—Imaris software (Bitplane™). Wheat leaves are in green color and fungal tissue is in blue; except for the fungal spore (green) due to lack of staining by Uvitex 2B owed to melamine presence in spore. Pathogen infection structures, including germ tube and appressorium, are observed on the leaf surface. Appressorium is observed over stomata.
10.1186/s13007-015-0096-0 3D view of wheat leaves infected with *Puccinia triticina* (16 hpi). The video shows seven-day-old seedling plants of susceptible cultivar Thatcher infected with *P. triticina*. Specimen was stained using the Uvitex 2B rapid staining method and imaged at 16 hpi using confocal microscopy and analyzed with Contour Surface Module—Imaris software (Bitplane™). Wheat leaves are indicated by green color and fungal tissue is indicated by blue; except for the fungal spore (green) due to lack of staining by Uvitex 2B owed to melamine presence in spore. Pathogen infection structures, including germ tube and appressorium, are observed on the leaf surface. Appressorium forms over stomata, penetration peg and sub-stomatal vesicle (SSV) formd in the sub-stomatal chamber, and primary infection hyphae is observed between the mesophyll cells. Haustoria mother cells (HMCs) develop from infection hyphae once in contact with mesophyll cells. Additional infection hypha develop from HMCs after the formation of first haustorium. Infection hyphae give rise to additional HMCs and haustoria when hypha come into contact with additional mesophyll cells.

## Abbreviations

*Pt*: *Puccinia triticina*
DPI: Days post inoculation
RH: Relative humidity
HPI: Hours post incubation
LCM: Laser capture microdissection
RNA: Ribonucleic acid
RT-qpcr: Reverse transcriptase quantitative polymerase chain reaction
PEN: Polyethylene naphthalate
Cq: Threshold cycle
